# Neutrophils and neutrophil extracellular traps in oral health and disease

**DOI:** 10.1038/s12276-024-01219-w

**Published:** 2024-05-01

**Authors:** Tae Sung Kim, Niki M. Moutsopoulos

**Affiliations:** Oral Immunity and Infection Section, National Institute of Dental and Craniofacial Research, National Institutes of Health, Bethesda, MD 20892 USA

**Keywords:** Mucosal immunology, Cell signalling

## Abstract

Neutrophils perform essential functions in antimicrobial defense and tissue maintenance at mucosal barriers. However, a dysregulated neutrophil response and, in particular, the excessive release of neutrophil extracellular traps (NETs) are implicated in the pathology of various diseases. In this review, we provide an overview of the basic concepts related to neutrophil functions, including NET formation, and discuss the mechanisms associated with NET activation and function in the context of the prevalent oral disease periodontitis.

## Neutrophils and NETs: Guardians and Culprits in Oral Mucosal Health and Disease

Neutrophils, the most abundant white blood cells, are essential for maintaining barrier tissue homeostasis and fighting infections. However, excessive neutrophil recruitment or activation can lead to tissue damage and inflammatory disease. Kim and Moutsopoulos review the roles of neutrophils, particularly focusing on Neutrophil Extracellular Traps (NETs) and their function in health and disease. Their review particularly focuses on functions of neutrophils and NETs within the oral mucosa, and explores mechanisms related to NET triggering and NET-mediated immunopathology in the setting of the oral mucosal disease, periodontitis.

This summary was initially drafted using artificial intelligence, then revised and fact-checked by the author.

## Introduction

Neutrophils play pivotal roles at mucosal barriers, including helping maintain tissue integrity, performing antimicrobial immune defense functions, and facilitating wound healing^1^. These abundant white blood cells serve as the first line of defense against microbial threats by effectively controlling infections and preserving mucosal integrity through processes such as phagocytosis, degranulation, and the release of neutrophil extracellular traps (NETs)^1^. At steady state and during infection, they eliminate pathogens and remove debris, and a rapid and effective response is necessary to promote wound healing^2,3^. However, excessive or dysregulated neutrophil responses are related to the pathogenesis of a wide variety of inflammatory, autoimmune, and obesity-related diseases^4^. Periodontitis is an inflammatory condition in which neutrophil function is critical^1,5^. Periodontitis is a common human inflammatory disease that affects the tissues supporting teeth. In this condition, disease-associated microbiota trigger inflammation in the mucosa surrounding the dentition and induce the destruction of soft tissues and supporting bone; in severe cases, periodontitis can lead to the loss of teeth^6^. Deficiencies in neutrophils, as well as the accumulation of neutrophils and NETs, have been implicated in different forms of periodontitis^1,5^. In this review, we provide an overview of the general functions and potential roles of neutrophils in periodontitis, with a particular focus on NETs and their role in oral mucosal immune homeostasis and inflammation.

## A primer for investigating neutrophil biology with a focus on neutrophil extracellular traps (NETs)

Neutrophils, which are the most abundant white blood cells in the body, are considered the cornerstone of innate immunity. Neutrophils originate in the bone marrow, mature, and typically become terminally differentiated before moving into the circulatory system. In the bone marrow, these cells are derived from hematopoietic stem cells and start as self-renewing hematopoietic stem cells that transform into multipotent progenitors (MPPs)^1^. These MPPs, in turn, differentiate into common myeloid progenitors (CMPs) and subsequently transform into granulocyte/monocyte progenitors (GMPs). GMPs exhibit limited proliferative potential but can differentiate into various cell types, including neutrophils, monocytes, dendritic cells, macrophages, eosinophils, basophils, and mast cells. Granulopoiesis, which marks the development of neutrophils, commences with CMPs and GMPs, leading to the production of proliferative pre-neutrophils that are characterized as CXCR4^+^ CXCR2^−^ cells. These pre-neutrophils then differentiate through various stages, including immature neutrophils (myelocytes, metamyelocytes, and band neutrophils), eventually reaching the mature neutrophil stage with the expression of CXCR2^7,8^. Through their differentiation and maturation stages, neutrophils acquire different types of granules, each containing specific contents that become critical for the different functions of the neutrophil. Neutrophils have at least four different types of granules^9^: (1) primary-azurophilic granules (e.g., elastase and myeloperoxidase (MPO)), (2) secondary-specific granules (e.g., iron-binding protein lactoferrin), (3) tertiary-gelatinase granules (e.g., matrix metalloproteinases (MMPs)) and (4) secretory vesicles.

**Fig. 1:**
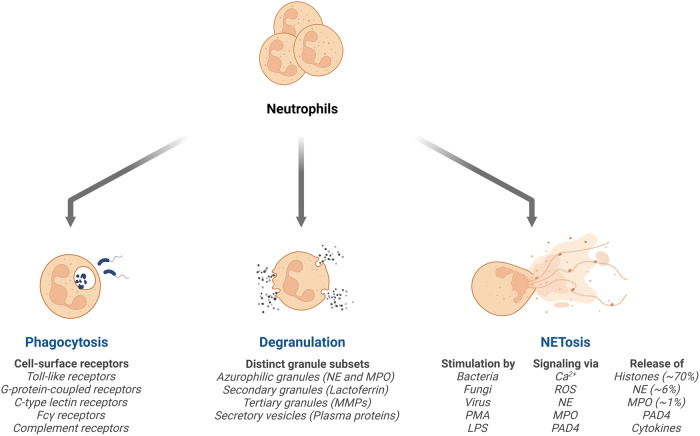
The innate antimicrobial defense mechanisms of neutrophils. Neutrophils mediate antimicrobial defense primarily through three major mechanisms: phagocytosis, degranulation, and the formation of neutrophil extracellular traps (NETs). Phagocytosis is triggered through a variety of cell surface receptors (major receptors indicated). Neutrophils also secrete a variety of enzymes and antimicrobial components, including those found in their granules (major types of neutrophil granules and contents are indicated). Finally, diverse stimuli (indicated) can activate intracellular pathways that mediate the formation and release of NETs.

Major neutrophil functions include phagocytosis, degranulation, and NET release (Fig. 1), all of which have been associated with the antimicrobial functions of the cell. Neutrophils eliminate both intra- and extracellular pathogens through phagocytosis, which is the process of engulfing and digesting foreign invaders such as bacteria, fungi, and cellular debris. Upon arrival at the sites of infection, neutrophils express a range of cell-surface receptors, including Toll-like receptors, G-protein-coupled receptors, C-type lectin receptors, Fcγ receptors, and complement receptors^10^. Microbial engagement of these receptors triggers phagocytosis, during which the neutrophils engulf pathogens within specialized vacuoles called phagosomes. Following engulfment, neutrophils employ diverse mechanisms to eliminate pathogens. These mechanisms include oxygen-dependent processes, commonly referred to as respiratory or oxidative bursts, as well as oxygen-independent methods that involve the action of lytic and proteolytic enzymes^11^. Upon activation, an NADPH oxidase complex produces a large amount of superoxide, which is rapidly broken down into hydrogen peroxide and other products, including hydroxyl radicals and oxygen, by superoxide dismutase. Reactive oxygen intermediates (ROIs) play an important role in killing bacterial and fungal pathogens. Oxygen-independent mechanisms rely on antimicrobial peptides and enzymes to facilitate the killing and degradation of ingested microbes^11^. In this manner, ingested microbes can be effectively eliminated by neutrophils. Additionally, neutrophils can mediate antimicrobial defense through neutrophil degranulation. Neutrophil degranulation refers to the process by which neutrophils release the contents of their granules, including chemicals, proteolytic enzymes such as elastase, MPO, and antimicrobial peptides (cathelicidins and defensins). This process allows neutrophils to combat pathogens during infections and regulate immune responses at sites of inflammation.

**Fig. 2:**
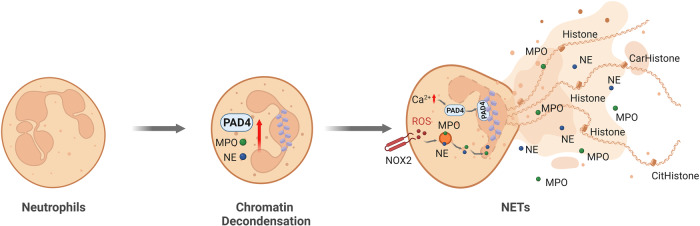
Lytic neutrophil extracellular trap (NET) formation. Various stimuli, including microbes, cytokines, PMA, platelets, monosodium urate (MSU) crystals, and nitric oxidase, can induce the formation of NETs. During this process, neutrophils undergo morphological changes to become rounded and have uniformly condensed chromatin. Neutrophils are activated to produce cytosolic ROS through the NOX2 complex. This activation prompts the release of neutrophil elastase (NE) and myeloperoxidase (MPO) from granules, followed by nuclear translocation of NE and MPO and the induction of chromatin decondensation. Subsequently, the nuclear envelope breaks down, leading to the fusion of DNA-containing vesicles with the plasma membrane. This cascade ultimately results in the formation of nuclear NETs. Additionally, elevated intracellular calcium levels induced by calcium ionophores can activate PAD4 independently of the NOX2 complex. PAD4 is translocated to the nucleus to drive histone citrullination/carbamylation and chromatin decondensation through a NOX2-independent mechanism.

Finally, neutrophils can also form structures called NETs, which are strand-like webs of decondensed chromatin that contain histones, over 30 components of granule proteins (including lactoferrin and cathepsins) and components of bactericidal activity enzymes (MPO and elastase). NETs, which were initially described by Brinkmann in 2004, function as bactericidal traps that effectively promote the elimination of extracellular bacteria^12^. The mesh-like NETs trap, neutralize, and eradicate a wide range of microorganisms, including bacteria (gram-positive and gram-negative bacteria, including *Staphylococcus aureus*)^12,13^, fungi (*Candida albicans*)^14^, viruses (human immunodeficiency virus (HIV)-1, respiratory syncytial virus (RSV))^15,16^, and parasites (*Toxoplasma gondii* and *Aspergillus fumigatus*)^17,18^, thereby playing a crucial role in preventing the dissemination of both bacterial and fungal pathogens. The release of NETs can be triggered by pathogen infections as well as host-derived components. NET formation typically begins with the activation of neutrophils in response to a variety of stimuli, including phorbol 12‑myristate 13‑acetate (PMA)^19,20^, cytokines (tumor necrosis factor-α and interleukin-8)^12^, nitric oxide^21^, high glucose^22^, activated platelets^23,24^, and complement^25^. NET formation is a multistep process that ultimately leads to chromatin decondensation, nuclear rupture, and NET release (Fig. 2). NET formation typically begins with plasma membrane rupture, termed lytic NETosis, although not all pathways result in cell lysis. Chromatin decondensation is a critical step that is regulated by different mechanisms. The Raf-MEK-ERK pathway plays a pivotal role in activating NOX2 (NADPH oxidase 2), which promotes NOX2-dependent NET formation in response to various stimuli, including PMA, activated platelets and different bacteria, such as *Helicobacter pylori* and *Toxoplasma gondii*. These stimuli can induce reactive oxygen species (ROS) production through NOX2 activation via the Raf-MEK-ERK pathway^17,26^. However, other stimuli, including calcium ionophores^20^^,^ nigericin^27^, ultraviolet (UV) irradiation^28^ and specific crystals^29^, induce NET formation, which is independent of NOX2 but dependent on mitochondrial ROS. Monosodium urate crystal (MSU)-induced NETs involve receptor-interacting protein kinase 1 (RIPK1), receptor-interacting protein kinase 3 (RIPK3), mixed lineage kinase domain-like pseudokinase (MLKL), and necroptosis-related signaling pathways^29^.

The formation of NETs involves multiple crucial steps^30,31^ (Fig. 2). One critical step is the production of ROS from the cytosol or mitochondria, which induces the release of neutrophil elastase (NE) and MPO from neutrophil granules. NE and MPO then migrate to the nucleus, where they contribute to chromatin decondensation^32^. In the nucleus, NE cleaves histones, thereby facilitating nuclear decondensation. MPO also binds to chromatin and collaborates with NE to further decondense chromatin, regardless of its enzymatic activity^32^. Another important step in NET formation is cytoplasmic granule dissolution and the activation of neutrophil proteases, where NE is released from neutrophil granules. While MPO plays a crucial role in facilitating NE release and activity, inhibiting the enzymatic activity of MPO merely results in a delay in NET formation rather than complete blockade. Histone modifications, particularly citrullination and/or carbamylation, are also involved in chromatin decondensation during NET formation^33^. Activated by an increase in intracellular calcium, peptidyl arginine deiminase 4 (PAD4) citrullinates histones in the nucleus, thereby reducing their positive charge and promoting chromatin decondensation. NETs were originally investigated for their antimicrobial activity^12,34^, and later, NET persistence was associated with inflammation and autoimmunity^30^. However, in certain situations, NETs exhibit anti-inflammatory effects through the capture and degradation of cytokines and chemokines^35^.

## Homeostatic functions of oral neutrophils

Neutrophils are present in several niches within the oral cavity. Under steady-state conditions, oral neutrophils are found in saliva^36^, oral mucosal tissues^37^ and gingival crevicular fluid^38^. Neutrophils mainly exit blood vessels and pass through the gingival junctional epithelium until they reach the periodontal pocket. Neutrophils can bind to endothelial cells through interactions with cell adhesion molecules such as selectins (E-selectin) and integrin receptors (β2-integrins) and migrate from the periodontal sulcus into the oral cavity. Several studies have suggested that neutrophils contribute to equilibrium and immunological homeostasis within the oral cavity. Within gingival crevicular fluids, neutrophils control the overgrowth of bacterial biofilms. Neutrophils are considered key cells that prevent infection and the invasion of bacteria into mucosal tissues^39^.

### Congenital neutrophil defects reveal the critical role of neutrophils in oral immunity

Genetic defects in neutrophil development and trafficking into tissues clearly demonstrate the critical role of neutrophils in oral immunity in humans. In fact, patients with congenital neutrophil defects present with very severe forms of periodontitis early in life and often lose their entire dentition by their teenage years or in early adulthood^40,41^.

**Fig. 3:**
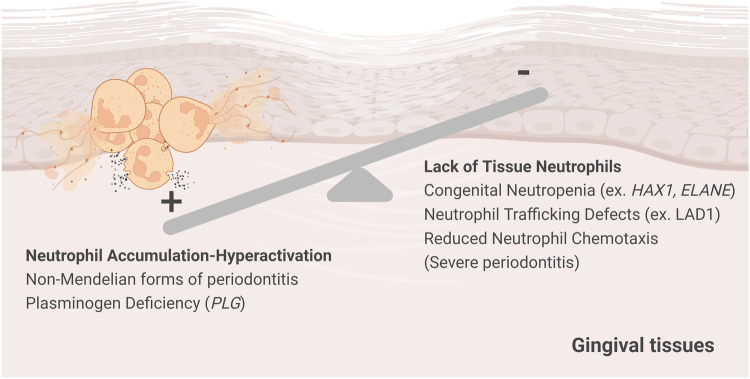
The balance of tissue neutrophils is essential for periodontal immunity. A balance in tissue neutrophils is essential for periodontal immunity. Indeed, both neutrophil deficiency and excessive neutrophil recruitment and activation have been linked to periodontitis. As such, patients with Mendelian genetic defects linked to neutropenia or defective neutrophil trafficking present with severe periodontitis at a young age. Moreover, excessive neutrophil activation and recruitment also leads to periodontitis both in the context of Mendelian disease (such as plasminogen deficiency) and in the setting of common forms of periodontitis.

Chédiak‐Higashi syndrome, Papillon‐Lefèvre syndrome, congenital neutropenia, and leukocyte adhesion deficiency I (LADI) are some examples of rare congenital, Mendelian forms of neutrophil disease (Fig. 3). In addition to Mendelian forms of periodontitis, early studies in patients with aggressive periodontitis reported a correlation between defective neutrophil chemotaxis and the severity of periodontal disease^42,43^.

Among the Mendelian forms of periodontitis, neutrophil development and trafficking disorders are most strongly associated with severe disease. Chediak-Higashi syndrome (CHS) is an autosomal recessive disorder caused by mutations in lysosomal trafficking regulator genes (LYST or CHS1)^44,45^. This syndrome is characterized by disrupted protein synthesis, which affects the storage and secretory functions of lysosomal granules in neutrophils and other cells, ultimately resulting in the abnormal fusion of lysosomes with the phagosome^46,47^. These mutations lead to decreased phagocytosis, defects in neutrophil trafficking and increased susceptibility to recurrent bacterial infections^48,49^. CHS has been associated with severe gingivitis or aggressive periodontitis. Earlier reports have noted that CHS patients frequently develop periodontal disease at a young age; this periodontal disease is characterized by significant and widespread deterioration of the tooth-supporting bone. This extensive bone loss results in tooth mobility and often leads to premature tooth loss between the ages of 15 and 20^50–52^. However, the precise molecular mechanisms that underlie periodontal tissue destruction in CHS are still not fully understood.

In other contexts, neutrophils may fail to reach gingival tissues, where they appear to play a crucial role in tissue homeostasis. Severe congenital neutropenia (SCN) encompasses a group of genetic disorders that are characterized by severe neutropenia, typically due to defects in neutrophil development^53^. SCN can be caused by mutations in various genes, with approximately 50% of cases involving mutations in the *ELANE* gene and approximately 10% involving mutations in the *HAX1* gene^54^. Numerous papers have described early-onset periodontitis in SCN patients, particularly in individuals carrying *ELANE* gene mutations^55,56^. *HAX1*, another gene associated with SCN, plays a pivotal role in mitochondrial regulation and apoptosis in neutrophils. Recent research suggests that mutations in *HAX1* may contribute to abnormal neutrophil function, thus exacerbating the risk of periodontitis. For example, in one case report, a 15-year-old adolescent girl developed early-onset periodontal disease that was characterized by gingival inflammation, aggressive bone destruction, tooth mobility, and recurrent oral infections^57^. Neutrophils can also fail to reach the gingiva due to recruitment defects, such as in LADI. LADI is considered a prototypic neutrophil defect linked to periodontitis. In LADI, neutrophils fail to exit circulation and transmigrate into tissues due to mutations in *ITGB2*, the gene that encodes the CD18 chain that is shared among all *β*2 integrins^58^. LADI is associated with periodontitis, particularly early-onset or severe periodontitis^59–61^. Finally, Papillon–Lefèvre syndrome (PLS) is a rare autosomal recessive genetic disorder caused by mutations in the cathepsin C (CTSC) gene^62^ and is associated with defective neutrophil function^63^. Periodontitis in individuals with PLS often begins during childhood or adolescence, potentially resulting in the premature loss of teeth^64,65^. Although the neutrophil-specific functions that are critical for oral/periodontal homeostasis have not been fully characterized, studies in rare diseases, particularly in LAD1, have shown that the mere presence of neutrophils is essential for regulating inflammation at the oral/gingival barrier^66,67^.

## Neutrophil accumulation and NET formation are evident in common and select genetic forms of periodontitis

Although the absence of tissue neutrophils has been clearly shown to predispose individuals to severe periodontitis at a young age, excessive neutrophil accumulation and activation have also been linked to periodontitis, suggesting that neutrophil balance is key for periodontal stability (Fig. 3). As such, while Mendelian neutropenia is linked to severe periodontitis at an early age, common (and select genetic) forms of periodontitis are associated with the accumulation/hyperactivation of neutrophil responses. However, neutrophil accumulation has long been observed in periodontitis lesions in humans and in animal models of disease^5,68^. Dysregulated secretion of the cytokine IL-17 has been linked to the exaggerated recruitment of neutrophils and to neutrophil-dependent bone destruction in periodontitis^69^. Developmental endothelial locus-1 (Del-1) is a protein that plays a crucial role in the regulation of neutrophil recruitment in inflammatory lesions in periodontitis. Indeed, in the absence of Del-1, there is excessive recruitment of neutrophils, leading to increased inflammation and the development of spontaneous periodontitis^70^. Importantly, the administration of human Del-1 in nonhuman primate models has demonstrated remarkable efficacy in alleviating inflammatory periodontal bone destruction^71^.

In addition to accumulation, the hyperactivation of neutrophils has been implicated in rapid tissue destruction, particularly in the context of aggressive forms of periodontitis^11,72,73^. The hyperactivation of neutrophils has been associated with increased neutrophil adhesion, enzyme secretion, and oxidative bursts. In periodontitis patients, increased levels of specific enzymes, such as β-glucuronidase^74^, myeloperoxidase^75^, and MMPs, including MMP-2, MMP-8, MMP-9, MMP-12, MMP-25, and MMP-26^76–82^, have been found in peripheral blood and gingival crevicular fluid. These enzymes are thought to play roles in tissue degradation and inflammation. Other studies have demonstrated an increase in oxidative bursts in patients with aggressive and chronic forms of periodontitis. Several studies have also reported higher levels of ROS production in patients with aggressive and chronic periodontitis^83–85^.

### Documentation of NET structures and associated components in patients with periodontitis

For more than a decade, numerous groups have reported on the presence of NETs in periodontitis lesions, as well as on the propensity of neutrophils from periodontitis patients to form NETs. Clinical studies related to NET detection in periodontitis patients have recently been comprehensively reviewed^86^. Early work from Vitkov et al. ^87^. using transmission and scanning electron microscopy, as well as cytology and confocal laser-scanning microscopy, demonstrated the presence of NETs within the gingiva biopsies and crevicular exudate of patients with chronic periodontitis. Following these initial observations, leaders in the field of periodontitis began to consider the possible roles of neutrophil NETosis in the pathogenesis of periodontitis^88^. Additional studies subsequently documented the presence of NETs and NET components within periodontal tissues and systemic circulation in periodontitis patients. For example, one study documented the presence of NET components such as citrullinated histone H3 (citH3), myeloperoxidase and NE within oral biofilms in experimental gingivitis. In another study, researchers utilized a flow cytometry NETosis assay to detect NET formation in oral neutrophil populations and found significantly increased NET formation in proinflammatory oral neutrophils from patients with periodontitis compared to those from healthy individuals^89^. Intriguingly, periodontal therapy was associated with a significantly decrease in NET production by peripheral blood mononuclear cells (PBMCs) from periodontitis patients, even though patient PBMCs were not shown to produce increased levels of NETs ex vivo^90^. Finally, in a cohort of patients with rheumatoid arthritis (RA), significantly increased levels of NETs and NET components with specific posttranslational modifications (carbmylated proteins, CarP) were detected in patients with periodontitis when compared to non-periodontitis RA patients. Furthermore, the levels of NETs and CarP were associated with increased periodontitis severity within that patient group^91^.

More recently, our group also performed a clinical study to assess the presence of NETs and associated components in lesions and the peripheral blood of patients with periodontitis. For these studies, we recruited patients with untreated and severe (stage III, IV) periodontitis, as well as age- and sex-matched healthy volunteers with pristine oral health^73^. All participants (healthy and periodontitis) were screened meticulously for known confounders associated with periodontitis, as well as NETosis, including smoking status, diabetes status, rheumatoid arthritis status, and any other known medical conditions. Participants were also medication naïve, including from the absence of antibiotics and anti-inflammatories for more than 3 months, per clinical study inclusion criteria (clinical trials.gov ID-NCT01568697). In this meticulously curated patient cohort, we quantified the NET complexes citH3-dsDNA and CarP-dsDNA (previously described as markers of carbamylated NETs^33,92^), as well as carbamylated histone H3/H4, at the site of disease (gingival crevicular fluid, GCF) and in the systemic circulation of patients and controls. We found that the levels of NET complexes, as well as citrullinated and carbamylated histones, were significantly elevated in the local lesions (GCFs) and blood of patients with severe periodontitis, as was measured based on periodontal bone destruction^73^; furthermore, these levels correlated with disease severity, even in the absence of confounding systemic disease. We hypothesize that this significant correlation between the levels of carbamylated NETs and the severity of periodontitis may have value when considering the utility of NETs as biomarkers in periodontitis; we currently lack biomarkers that can predict periodontitis initiation and progression. Such biomarkers can be particularly important for the early detection of disease and for predicting disease progression in patients, ideally leading to personalized prevention and/or timely and targeted therapeutic intervention.

### NETs may play homeostatic roles in periodontal immunity

Accumulating evidence suggests that NETs play a protective role in the oral cavity, particularly in relation to their antimicrobial properties. Recent work has demonstrated that at least 19 oral bacteria associated with periodontitis have the capacity to induce NET formation^93^. Furthermore, the antimicrobial properties of DNA and peptides in NETs and their potential for killing periodontal bacteria have been well documented^87,94,95^. NETs can trap and neutralize harmful bacteria that might otherwise contribute to periodontal diseases, gingivitis, and periodontitis. Additionally, the abundance of NETs in gingival tissue samples and in the crevicular exudate of periodontitis patients has been well documented. Oral NETs trap and prevent the adhesion and invasion of crevicular bacteria, suggesting a novel defense mechanism that leads to the clearance of bacteria in chronic periodontitis^87^. Furthermore, NETs have been shown to interact with salivary components, thereby actively participating in enhancing the overall antimicrobial properties of the oral environment^96^. Additionally, NETs have been shown to play important roles in wound healing. They act as a scaffold for tissue repair and concurrently capture potential pathogens that might hinder the healing process^96^, thereby contributing to an efficient and protected wound-healing environment, which can be particularly important in an area that is under constant threat, such as the oral mucosa. In summary, NETs are associated with key immunoprotective defenses at the oral barrier, and future in vivo evidence in animal models or patients with NETosis defects will further strengthen our understanding of the role of NETs in oral mucosal homeostasis.

## NETs are involved in the pathogenesis of various inflammatory conditions

Aberrant NET formation has been associated with the pathogenesis of various diseases, ranging from autoimmunity (such as systemic lupus erythematosus, SLE and rheumatoid arthritis [RA]) to sepsis and infectious diseases (ex. COVID-19 infection). Below, we will highlight the role of NETs in various disease pathologies in an effort to review potential NET-associated functions that could be relevant to their inflammatory roles in oral mucosal disease.

### Autoimmune diseases: SLE and RA

Aberrant NET formation and defective NET degradation are observed in various diseases and contribute to the pathogenesis of various autoimmune pathologies. In certain autoimmune disorders, NETs can contribute to the generation of autoantibodies by exposing the host to self-antigens or modified autoantigens, such as those associated with RA. As such, in RA, antibodies against citrullinated protein antigens (ACPAs) from NETs are well recognized as prognostic biomarkers and are characteristic of disease^97^. In SLE, immune complexes that contain NET autoantigens coupled with autoantibodies contribute to vascular inflammation and endothelial dysfunction. Furthermore, glomerulonephritis in SLE has been associated with NETs, which play a role in renal tissue damage^98^.

### Sepsis

The association between NETs and sepsis pathogenesis has been demonstrated through numerous studies in patients and animal models of disease. Overall, NET levels are measured based on circulating free DNA (cf.-DNA)/NETs; in addition, NETs are generated during sepsis and are associated with disease severity^99^. One clinical study examining sepsis development and mortality after multiple traumatic events reported that elevated levels of cf.-DNA in the early stages correlated with subsequent sepsis, multiorgan dysfunction, and death^100^. In another investigation of patients diagnosed with septic shock, researchers explored NET-associated MPO-DNA levels in plasma. Their results showed a significant increase in plasma MPO-DNA levels on days 1, 3, and 7 compared to patients in the healthy control group. Elevated MPO-DNA levels on days 3 and 7 were closely associated with a higher 28-day mortality rate, suggesting that excessive NET formation plays a role in the pathogenesis of septic shock^101^. NETs have been implicated in host tissue damage during sepsis^102^. Higher concentrations of cf.-DNA were detected in septic patients and were associated with the severity of sepsis and organ dysfunction. In a methicillin-resistant *Staphylococcus aureus* (MRSA) sepsis model, NETs were implicated in vascular wall damage and subsequent liver tissue damage. Importantly, the inhibition of NET formation using genetic models (such as those with protein arginine deiminases 4 deficiency (*padi4*^-/-^) or neutrophil elastase deficiency (*elane*^-/-^)) effectively prevented host tissue damage^102^. In a mouse model of polymicrobial sepsis induced by cecal ligation and puncture (CLP), model mice had elevated serum levels of NETs compared to those in control mice. Treatment with recombinant human deoxyribonuclease (rhDNase) effectively reduced NET formation, particularly when combined with antibiotic therapy, thereby partially alleviating organ damage. Furthermore, administration of DNase to improved survival rates, and these experiments provided evidence that delayed administration of DNase may protect against experimental sepsis, in contrast to the effects of early administration^103^.

### Atherosclerosis

A growing body of evidence has highlighted the importance of NETs in atherosclerosis and related conditions, suggesting the potential of NETs as diagnostic or prognostic indicators. Atherosclerosis arises from a persistent inflammatory process primarily driven by lipids and involves vascular and immune cells^104^. Hyperlipidemia-induced neutrophilia has been confirmed to be positively correlated with atherosclerosis in humans^105^. Experiments in which ApoE- or low-density lipoprotein receptor-deficient mice were fed a high-fat diet confirmed the significant role of neutrophils in plaque development^106^. NETs within the atherosclerotic environment have been observed both in ApoE-deficient mice and in patients with atherosclerosis^107^. Examination of human atherosclerotic lesions directly demonstrated the presence of neutrophils, NETs^108^ and MPO^109,110^ as likely inflammatory inducers within the lesion. NETs have been shown to induce lytic cell death in smooth muscle cells within atherosclerotic lesions, and histones derived from NETs reduce plaque stability^111^. Furthermore, neutrophil infiltration is correlated with the inflammatory state of lesions prone to rupture; the levels of surrogate marker dsDNA and chromatin are independently associated with the severity of coronary atherosclerosis and adverse cardiovascular events^112,113^.

### Coronavirus disease 2019 (COVID-19)

More recently, NETs have been implicated in the pathology and tissue damage associated with SARS-CoV-2 infection. Autopsy findings support the hypothesis that the ability of neutrophils to form NETs may contribute to organ damage and mortality in patients with COVID-19^114^. Numerous studies support the idea that NET formation in various organs can contribute to organ damage and mortality in patients with COVID-19. Specifically, lung infiltration of neutrophils was observed in autopsy specimens from deceased COVID-19 patients. Cell-free DNA, MPO-DNA and citH3 were also detected in COVID-19 patients. These components are released by and associated with the severity of COVID-19 symptoms^115^. Therefore, targeting NETs in COVID-19 patients has been proposed as a tentative strategy to reduce disease severity and the impact of neutrophil-induced multiorgan damage during severe COVID-19^116^ infection.

## Extracellular histones in inflammation and disease

Histones serve as structural units that tightly wrap and condense DNA within the nucleus, thus shaping the structure of chromosomes. Histones play a vital role in orchestrating and regulating gene expression^117^. However, under certain circumstances, histones can translocate from the nucleus to the extranuclear space, where they assume different roles. In this extranuclear context, histones are cytotoxic and can act as “danger signals” or “DAMPs” (damage-associated molecular patterns^118,119^). Recent work from the Papayannopoulos laboratory revealed that histones and DNA synergize to induce extracellular sublethal signaling^120^. These authors showed that histones can bind and activate TLR4, thereby mediating proinflammatory responses. As such, excessive histone release can promote inflammation. In the following section, we will briefly discuss conditions linked to extracellular histone release and the associated mechanisms of histone-mediated inflammation and pathology.

### Infectious diseases

#### Sepsis

Extracellular histones have been shown to contribute to endothelial dysfunction, organ failure, and mortality in models of sepsis. In fact, treatment with histone-deleting antibodies effectively improved survival in mouse models of sepsis^118^. In a mouse model of LPS-induced sepsis, the authors observed an increase in the serum levels of histone H3, which was found to be associated with the severity of disease pathogenesis. Additionally, the serum concentration of citH3 has emerged as a critical indicator of disease severity. Treatment with suberoylanilide hydroxamic acid (SAHA), a histone deacetylase inhibitor, not only reduced the serum levels of histone H3 and citH3 but also improved the survival rate. These findings suggest that citH3 could serve as a protein biomarker for the early diagnosis of severe sepsis or septic shock, positioning it as a key prognostic factor for sepsis^121^. In a subsequent study, researchers focused on the role of citH3 and histone deacetylase (HDAC) inhibitors in septic shock. Using a mouse model of septic shock, they examined the impact of HDAC and citH3 inhibitors on mouse survival. This study revealed that both HDAC inhibition and citH3 neutralization suppressed citH3 production and improved the survival rate of mice. These findings suggest that citH3 could be useful in the early diagnosis and treatment of septic shock and suggest its potential as a novel therapeutic target^122^.

### Peritonitis

Histones released from necrotic cells play a pivotal role in various inflammatory conditions, including peritonitis. Histones activate the NLRP3 inflammasome, a multiprotein complex that regulates the release of caspase-dependent cytokines such as IL-1β and IL-18. Exogenous histones induce neutrophil recruitment and IL-1β release through an NLRP3-caspase-1-dependent mechanism. Importantly, treatment with H4-neutralizing antibodies ameliorates the severity of histone-induced peritonitis^123^.

### Neurodegenerative diseases

Extracellular histones have been implicated in several neurodegenerative diseases, including Alzheimer’s disease (AD), Parkinson’s disease (PD), prion disease and amyotrophic lateral sclerosis (ALS). In AD, histone H1 is linked to extracellular amyloid plaques^124^. Histone detection within the brain tissue of AD patients also revealed that histone levels are primarily increased in regions associated with disease pathology^125^. Furthermore, in both prion disease and AD, nonnuclear histone H1 expression is upregulated in neurons and astrocytes. Additionally, extracellular histones were detected in a transgenic mouse model of ALS^126^. Extracellular histone/nucleosome release has been reported to occur during ischemic stroke/reperfusion injury. Similar to those in stroke patients, elevated levels of circulating nucleosomes and DNA were observed in mice following ischemic stroke. Treatment targeting the components of circulating chromatin improved stroke outcomes. Conversely, injection of histones in vivo in mouse models amplified stroke symptoms with an increase in infarct volumes, which could be reduced by anti-histone H4 antibody treatment^127^.

### RA

Recent studies have also revealed the role of extracellular histones in RA. Histones have recently been shown to promote osteoclast activation, and histones with particular posttranslational modifications have been associated with erosive bone disease in individuals with RA^33,128^. Indeed, NETs with carbamylated proteins, including histones H3 and H4, are associated with the development of autoantibodies against carbamylated (cNET) antigens in RA patients. These cNET antibodies were associated with bone erosion in RA patients. Similar findings were also observed in a mouse model mimicking human RA^33^. Carbamylated histones within NETs enhance pathogenic immune responses and contribute to bone destruction; this result explains the link between anti-CarP antibodies and erosive arthritis in patients with RA^33^. Further studies revealed that osteoclast formation mediated by NETs depends on TLR4 signaling and NET-associated proteins, particularly carbamylated histones H3 and H4, as well as NE. These proteins and signaling pathways play a crucial role in promoting osteoclast formation via NETs^128^.

## Periodontitis: insights into the activation and function of NETs in periodontitis

### Microbial triggering

As discussed above, NET formation can be triggered via numerous stimuli, with microbial stimuli being a major mechanism for induction, particularly during infection and at barrier sites. As such, the ability of the oral microbiota to trigger NETosis has been evaluated in vitro. One study evaluated the capacity of 19 oral bacteria to induce NET formation in human PBMCs. The authors reported that various oral microbes could trigger NET formation with specific bacteria, such as *Propionibacterium acnes*, *Veillonella parvula*, and *Streptococcus gordonii*, inducing higher levels of NETs than other oral bacterial species that were

evaluated^93^. Moreover, additional in vitro studies have reported that bacteria associated with periodontitis can trigger the formation of NETs. *Porphyromonas gingivalis (P. gingivalis*), a classic periodontitis-associated microbe, was shown to trigger significant NET formation. *P. gingivalis*-induced NET formation is primarily attributed to the capacity of specific microbial proteins called gingipains to trigger NET induction^129^. Another periodontal microbe, *Fusobacterium nucleatum*, was found to trigger NET formation*. F. nucleatum* was shown to activate neutrophils and promote citH3 production in human neutrophils^130^. Such in vitro observations provide interesting insights into the role of oral and periodontal microbial communities in neutrophil activation and NET formation.

### Fibrin is a key regulator of neutrophil effector function and NETosis in the oral mucosa

Beyond microbial triggering, recent work from our laboratory revealed that the accumulation of extravascular fibrin becomes a scaffold for the retention of neutrophils within oral mucosal tissues and facilitates the activation of neutrophil effector functions, including ROS production and the release of NETs^131,132^. Neutrophils bind to fibrin through the αMβ2 integrin receptor, and this engagement promotes the release of NETs.

Fibrin, which is the key component of hemostasis, is released from the circulation into tissues in response to injury, infection and/or inflammation as capillary vessels become leaky or rupture. This accumulation of fibrin becomes critical for the formation of a hemostatic plug that prevents excessive bleeding. However, fibrin should be removed after hemostasis to allow wound healing to occur. Defective fibrinolysis, typically due to the absence or reduction of the key enzyme plasminogen, leads to the accumulation of fibrin within tissues, especially at mucosal sites, leading to widespread mucosal immunopathology in humans and experimental models of plasminogen deficiency^133,134^. In fact, one of the hallmarks of plasminogen deficiency in humans is ligneous periodontitis, which is characterized by fibrin accumulation in oral tissues that leads to severe bone loss around teeth as early as childhood. Importantly, within tissues, neutrophils bind to fibrin through the αMβ2 (Mac-1) integrin receptor. Binding to fibrin increases neutrophil retention and the activation of neutrophil effector functions, such as ROS production and NET release^131^. Importantly, this fibrin-specific binding of myeloid cells is responsible for periodontal destruction in the context of plasminogen deficiency. NET formation is a key mediator of plasminogen deficiency pathology, as timely removal of NETs using DNase I treatment significantly protects against periodontal bone loss in experimental models.

### NETs and extracellular histones mediate periodontal pathology

**Fig. 4:**
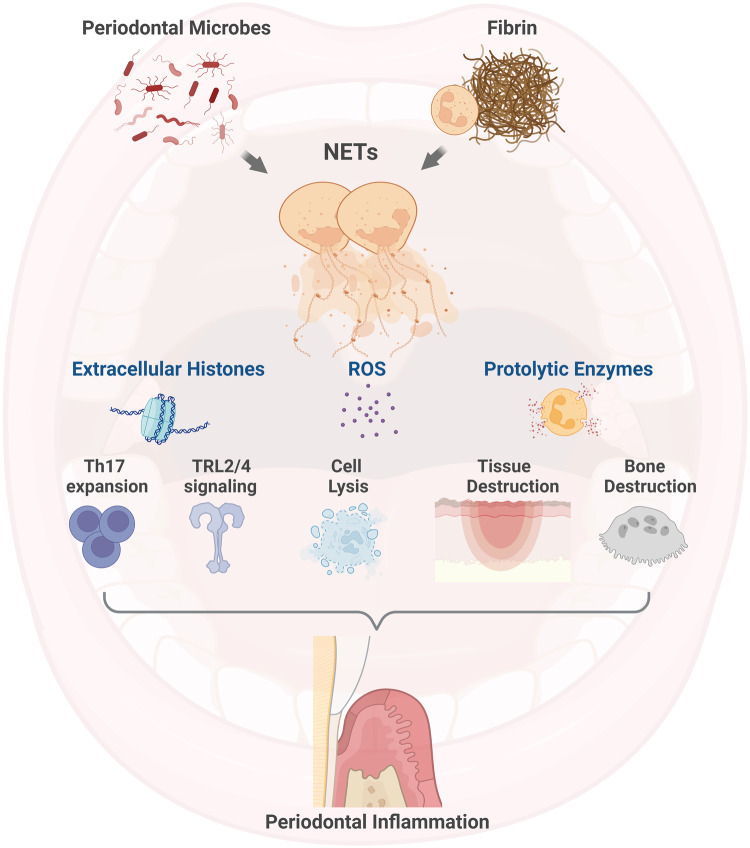
The activation and function of NETs in periodontitis. In periodontitis, NET formation can be triggered by oral microbes. Furthermore, the engagement of neutrophils with extravascular fibrin within mucosal tissues has been shown to potentiate NET formation. NET formation can trigger periodontitis in multiple ways through the inflammatory actions of extracellular histones, reactive superoxide species (ROS) and the release of proteolytic enzymes, all of which are capable of mediating cell lysis; NET formation can also trigger periodontitis via the engagement of known pathways linked to the pathogenesis of periodontitis.

Interestingly, NETs are pathogenic triggers of periodontal bone loss even in the absence of fibrinolysis defects, suggesting that this pathway plays a role beyond plasminogen deficiency-associated periodontitis^73^. Recent work from our laboratory using plasminogen-sufficient mice demonstrated that NETs promote oral mucosal inflammation and periodontal bone destruction in the absence of defective fibrinolysis. Indeed, the inhibition of NETosis in multiple genetic and pharmacologic models, as well as timely discontinuation of NETosis through DNase I treatment, significantly prevented experimental periodontitis in a ligature-induced periodontitis model in C57BL6 mice. By elucidating the mechanisms by which NETs trigger inflammation and bone destruction in periodontitis, we revealed that extracellular histones, which are major NET components, are key mediators of disease pathogenesis. Indeed, the inhibition of extracellular histones using antibody treatments against histones H3 and H4 reduced disease pathology to a comparable extent to the inhibition of NETosis. We found that removing either NETs or extracellular histones prevents the induction of key inflammatory pathways linked to periodontitis. The accumulation of Th17 cells, which trigger experimental periodontitis^69,135^, was significantly reduced when extracellular histones were blocked. Although in vivo data conclusively documenting the effect of NETs and histones on Th17 expansion are not available, there is substantial evidence to suggest that extracellular histones play a direct role in Th17 expansion. As such, we showed that NETosis occurs early in disease response, just prior to Th17 accumulation in the model, and we observed the spatial association of neutrophil NETs with T cells in disease lesions and found that Th17 cells in periodontitis express TLR2, a known ligand of extracellular histones^1^. However, NETs and extracellular histones can play pleotropic roles within disease lesions. NET components include a plethora of proinflammatory mediators, including various proteases that can trigger inflammation and matrix degradation^136–138^. Histones can also trigger inflammation in various ways, ranging from the induction of cell death to the activation of TLR2 and TLR4 signaling in multiple cell types^139–142^. The proposed functionalities of NETs and histones in periodontitis are summarized in Fig. 4.

## Concluding remarks

Overall, in this review, we discuss basic concepts related to NET formation and its diverse roles in the setting of inflammatory disease, with a particular focus on the oral mucosal disease periodontitis.
